# Decreased Susceptibility to Azithromycin in Clinical *Shigella* Isolates Associated with HIV and Sexually Transmitted Bacterial Diseases, Minnesota, USA, 2012–2015

**DOI:** 10.3201/eid2604.191031

**Published:** 2020-04

**Authors:** Dana Eikmeier, Pamela Talley, Anna Bowen, Fe Leano, Ginette Dobbins, Selina Jawahir, Annastasia Gross, Dawn Huspeni, Allison La Pointe, Stephanie Meyer, Kirk Smith

**Affiliations:** Minnesota Department of Health, St. Paul, Minnesota, USA (D. Eikmeier, F. Leano, G. Dobbins, S. Jawahir, A. Gross, D. Huspeni, A. La Pointe, S. Meyer, K. Smith);; Centers for Disease Control and Prevention, Atlanta, Georgia, USA (P. Talley, A. Bowen)

**Keywords:** Shigella, bacteria, shigellosis, men who have sex with men, MSM, azithromycin, decreased susceptibility to azithromycin, DSA, antimicrobial resistance, HIV, sexually transmitted diseases, STDs, infections, sexually transmitted infections (STIs), enteric infections, Minnesota, United States

## Abstract

Shigellosis outbreaks caused by *Shigella* with decreased susceptibility to azithromycin (DSA-*Shigella*) among men who have sex with men (MSM) have been reported worldwide. We describe sexual health indicators and antimicrobial drug resistance for shigellosis cases in Minnesota, USA. We analyzed a sample of isolates received during 2012–2015 and cross-referenced cases with the Minnesota Department of Health Sexually Transmitted Disease Database to ascertain patients’ HIV status and recent chlamydia, gonorrhea, and syphilis infections. Of 691 *Shigella* isolates, 46 (7%) were DSA-*Shigella*; 91% of DSA-*Shigella* patients were men, of whom 60% were living with HIV. Among men, those with DSA-*Shigella* infection had greater odds of living with HIV, identifying as MSM, or having a recent diagnosis of a sexually transmitted disease. DSA-*Shigella* was associated with MSM, HIV infection, and recent sexually transmitted disease. To decrease spread of DSA-*Shigella*, interventions targeted at communities at high risk are needed.

*Shigella* is an enteric bacterial pathogen that causes diarrhea (sometimes bloody), fever, and cramps (*1*). An estimated 500,000 *Shigella* infections occur annually in the United States (*2*). *Shigella* transmission is fecal–oral; it is easily spread person-to-person because of a low infectious dose. Outbreaks are most frequently documented in childcare settings but have also been reported among men who have sex with men (MSM) (*3*–*7*). *Shigella* infections are typically self-limiting, but treatment is recommended for patients with severe illness or underlying immunocompromising conditions (*1*,*8*). Antimicrobial drug treatment might shorten illness duration and is often used in childcare-associated outbreaks to prevent secondary transmission (*1*,*8*). When *Shigella* antimicrobial drug susceptibilities are unknown (e.g., when empiric therapy is started before culture and sensitivity results are available) or if the isolate is resistant to ampicillin and trimethoprim/sulfamethoxazole, oral treatment options include ciprofloxacin or azithromycin (*1*). However, fluoroquinolones, including ciprofloxacin, are generally avoided for treatment in children because of the risk for musculoskeletal damage (*9*).

The emergence of *Shigella* with decreased susceptibility to azithromycin (DSA) has been reported in Asia, Europe, North America, and Oceania (*10*–*17*). Local outbreaks and intercontinental sexual transmission of DSA-*Shigella* have been observed among MSM (*10*,*15*,*18*–*20*). Co-infection with other enteric pathogens and sexually transmitted diseases (STDs) have also been reported among MSM (*20*–*24*).

In 2016, the Clinical and Laboratory Standards Institute (CLSI) defined epidemiologic cutoff values for azithromycin resistance in *S. sonnei* or *S. flexneri* for the first time (*25*). Previously, the National Antimicrobial Resistance Monitoring System (NARMS) had documented DSA (azithromycin MIC >16 µg/mL) among *Shigella* isolates*;* during 2011–2015, DSA prevalence increased from 0.9% to 6.1% among *S. sonnei* isolates and from 12.1% to 32.9% among *S. flexneri* isolates submitted to NARMS (*26*). The Minnesota Department of Health (MDH) Public Health Laboratory (PHL) began testing 10% of *Shigella* isolates for DSA in 2010 and found such an isolate in 2013. As a result, we began more extensive testing of isolates from 2012 onward to characterize the emergence of DSA-*Shigella*. The purpose of this study was to describe the relationship between the emergence of DSA-*Shigella* in Minnesota and HIV and bacterial STDs.

## Materials and Methods

In Minnesota, all *Shigella* infections must be reported to MDH. In addition, clinical laboratories must submit isolates or clinical specimens to the MDH PHL. Phone interviews with patients are attempted by using a standard questionnaire.

We analyzed all shigellosis cases in Minnesota residents who had a sample collected during January 1, 2012–December 31, 2015, that was culture-confirmed by the MDH PHL. Patients with >1 isolate were considered to have a new infection if specimens were collected >90 days apart or were different *Shigella* species. We collected information by telephone interview on demographics, travel history, children in the household, contact with a childcare facility, ill contacts, symptom history, and antimicrobial drug treatment. During 2012–2014, sexual activity in the week before onset was noted on the case report form when reported by the patient, but it was not routinely collected.

In 2015, sexual exposure questions (“in the 7 days before your onset of illness did you have any sexual contact with a male; did you have any sexual contact with a female?”) were added to the *Shigella* questionnaire for adult patients. Case name and date of birth were linked with the MDH STD, HIV, and Tuberculosis Section database to obtain HIV, chlamydia, gonorrhea, and syphilis test results, and antimicrobial drug treatment for such infections occurring during the 12 months before each *Shigella* isolation. Risk factors for HIV infection, such as identifying as an MSM during an HIV surveillance interview, were obtained for persons living with HIV (PLWH). Children were defined as persons <18 years of age. Recent STD was defined as chlamydia, gonorrhea, or syphilis infection in the 12 months before *Shigella* isolation.

We performed antimicrobial susceptibility testing by using the modified Kirby-Bauer disk diffusion method on all *Shigella* isolates collected during 2013 and 2014. We also performed antimicrobial susceptibility testing on the following isolates collected during 2012 and 2015: all *S. flexneri* isolates, all isolates from international travelers and adults, 75% of pediatric outbreak isolates, and 30% of the remaining sporadic pediatric isolates. The antimicrobial susceptibility testing panel included 8 classes of antimicrobial drugs: aminoglycosides (gentamicin, streptomycin), cephems (cephalothin, ceftriaxone), folate-pathway inhibitors (sulfisoxazole, trimethoprim/sulfamethoxazole), macrolides (azithromycin), penicillins (ampicillin), phenicols (chloramphenicol), quinolones (ciprofloxacin, nalidixic acid), and tetracylines (tetracycline). Susceptibility was classified by using CLSI guidance, including classifying DSA in *S. flexneri* as a <15 mm zone of inhibition for a disk containing 15 µg of azithromycin (BD BBL Sensi-Disc; Becton Dickinson, https://www.bd.com) (*25*). Because CLSI guidelines do not include disk diffusion epidemiologic cutoff values for use with *S. sonnei*, we conservatively defined DSA as no zone of inhibition (6 mm) by disk diffusion using disks containing 15 µg of azithromycin. A sample of isolates with DSA was submitted to NARMS for further characterization by PCR for the macrolide resistance genes *mph*A and *erm*B. Multidrug resistance (MDR) was defined as resistance to >3 antimicrobial drug classes and clinical resistance as resistance to >1 of the major antimicrobial drug classes (cephems, folate-pathway inhibitors, macrolides, penicillins, quinolones).

We compiled descriptive and summary statistics by using SAS version 9.4 (SAS Institute, https://www.sas.com). We analyzed categorical variables by using Fisher exact or χ^2^ tests as appropriate and compared medians by using 2-sided Wilcoxon rank-sum tests. Statistical significance was set at α<0.05.

## Results

**Figure 1:**
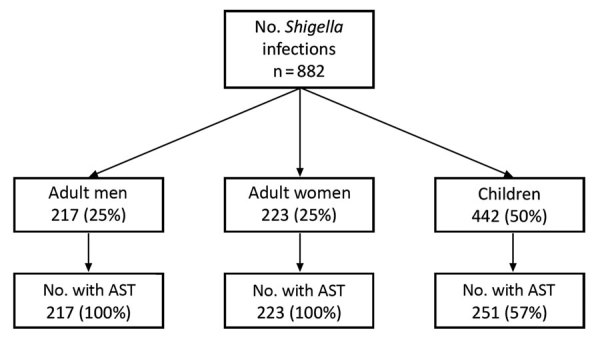
Number of *Shigella* isolates for which AST was conducted, by demographic group among isolates received at the Minnesota Department of Health, 2012–2015. AST, antimicrobial susceptibility testing.

During 2012–2015, a total of 882 infections in 878 patients were confirmed as *Shigella* by the MDH PHL. Among the 882 infections, 750 (85%) were caused by *S. sonnei*, 125 (14%) by *S. flexneri*, 5 (0.6%) by *S. boydii*, and 2 (0.2%) by *S. dysenteriae*. Half (n = 442) of the infections were in children, 223 (25%) were in adult women, and 213 (24%) were in adult men (Figure 1).

### Antimicrobial Drug Resistance

**Figure 2:**
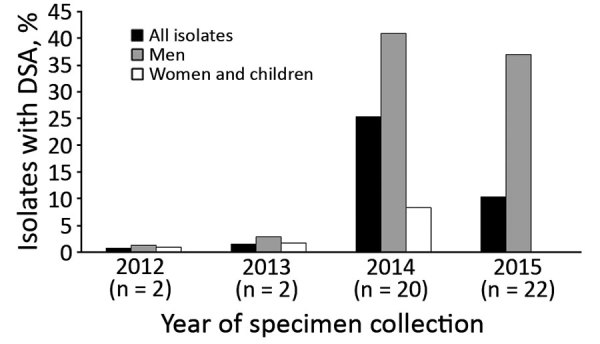
Percentage of 46 clinical *Shigella* isolates tested at the Minnesota Department of Health that had DSA, by year and demographic group, 2012–2015. DSA, decreased susceptibility to azithromycin.

Antimicrobial susceptibility testing was performed for 691 (78%) isolates; 46 (7%) isolates had DSA (24 [4%] of 559 *S. sonnei* isolates, 21 [17%] of 125 *S. flexneri* isolates, and 1 [20%] of 5 *S. boydii* isolates) (Table 1; Figure 1). Two DSA-*Shigella* isolates each were collected during 2012 and 2013; the remaining 42 isolates were collected during 2014 (*S. sonnei*, 16; *S. flexneri*, 4) and 2015 (*S. sonnei*, 7; *S. flexneri*, 15) (Figure 2).

**Table 1:** Frequency of azithromycin zone of inhibition for *Shigella* isolates by species, Minnesota, USA, 2012–2015*

Inhibition zone, mm	No. (%) isolates			
	*S. sonnei*, n = 559	*S. flexneri*, n = 125†	*S. boydi*, n = 5	*S. dysenteriae*, n = 2
6	24 (4)	18 (14)	1 (20)	0
8	0	1 (0.8)	0	0
12	0	1 (0.8)	0	0
14	0	1 (0.8)	0	0
15	1 (0.2)	0	0	0
17	2 (0.4)	0	0	0
18	3 (1)	0	0	0
19	5 (1)	2 (2)	0	0
20	23 (4)	4 (3)	0	0
21–25	442 (79)	48 (38)	2 (40)	1 (50)
26–30	58 (10)	44 (35)	2 (40)	0
>30	1 (0.2)	6 (5)	0	1 (50)

*Decreased susceptibility to azithromycin is defined as no zone of inhibition (6 mm) by disk diffusion using a disk containing 15 µg of azithromycin. †Decreased susceptibility to azithromycin is defined as a <15 mm zone of inhibition with a disk containing 15 µg of azithromycin.

Isolates with resistance to >1 of the 8 classes of antimicrobial drugs tested were observed among all sex and age groups (Table 2). After we excluded international travelers, we found that similar proportions of isolates from children and women were resistant to each of the antimicrobial drug classes except for folate-pathway inhibitors (children 57% resistant vs. women 40% resistant; p<0.001) and phenicols (children 9% resistant vs. women 3% resistant; p = 0.01). Isolates from men had a higher prevalence of resistance to all drug classes except for cephems and aminoglycosides and were more likely to have clinical resistance (Table 2).

**Table 2:** Antimicrobial drug class resistance for *Shigella* isolates, by patient sex, age, and international travel status, Minnesota, USA, 2012–2015*

Characteristic	Total, n = 691	Known international travel, n = 69	Men, n = 194†	Children and women, n = 428‡	OR (95% CI)§	p value
Antimicrobial drug class (drug)						
Aminoglycoside (GEN, STR)	660 (96)	61 (88)	191 (98)	408 (95)	3.1 (0.9–10.6)	0.07
Cephem (CRO, CEF)	13 (2)	2 (3)	3 (2)	8 (2)	0.8 (0.2–3.1)	1.0
Folate-pathway inhibitor (SUL, SXT)	416 (60)	56 (81)	148 (76)	212 (50)	3.3 (2.2–4.8)	<0.001
Macrolide (AZT)	46 (7)	3 (4)	42 (22)	1 (0)	118.0 (16.1–864.7)	<0.001
Penicillin (AMP)	162 (23)	27 (39)	74 (38)	61 (14)	3.7 (2.5–5.5)	<0.001
Phenicol (CHL)	76 (11)	22 (32)	26 (13)	28 (7)	2.2 (1.3–3.9)	0.01
Quinolone (CIP, NAL)	52 (8)	23 (33)	23 (12)	6 (1)	9.5 (3.8–23.6)	<0.001
Tetracycline (TET)	257 (37)	60 (87)	124 (64)	73 (17)	8.6 (5.9–12.7)	<0.001
Class resistance						
No resistance detected	6 (1)	2 (3)	0 (0)	4 (1)	NA	0.32
≥3 classes	266 (38)	60 (87)	130 (67)	76 (18)	9.4 (6.4–13.9)	<0.001
Clinical resistance¶	486 (70)	66 (96)	173 (89)	247 (58)	6.0 (3.7–9.9)	<0.001

*Values are no. (%) resistant except as indicated. Adults are persons >18 years of age; children are persons <18 years of age. AMP, ampicillin; AZT, azithromycin; CHL, chloramphenicol; CEF, cephalothin; CIP, ciprofloxacin; CRO, ceftriaxone; GEN gentamicin; NA, not applicable; NAL, nalidixic acid; OR, odds ratio; STR, streptomycin; SUL, sulfisoxazole; SXT, trimethoprim/sulfamethoxazole; TET, tetracycline. †Total excludes 23 known international travelers. ‡Total excludes 46 known international travelers. §Comparison of men with children and women. Reference group is children and women. ¶Resistance to >1 of the following antimicrobial drug classes: cephem, folate-pathway inhibitor, macrolide, penicillin, quinolone.

Forty-two (91%) of the 46 DSA-*Shigella* infections were in men; among the other 4 infections, 1 case in 2012 and 1 case in 2013 were in children and 2 cases in 2014 were in women (Figure 2). In comparison, only 175 (27%) azithromycin-susceptible infections were in men (odds ratio [OR] 28.2, 95% CI 10.0–79.8; p<0.001).

Three men had multiple *Shigella* infections. One patient had an azithromycin-susceptible *S. sonnei* infection in 2013 and a DSA *S. flexneri* 3b infection in 2015; 1 patient had a DSA *S. flexneri* 3b infection in 2015 and an azithromycin-susceptible *S. sonnei* infection that had a specimen collection date 66 days later; and 1 patient had an azithromycin-susceptible *S. sonnei* infection in 2012, an azithromycin-susceptible *S. flexneri* 3a infection in 2014, and a DSA *S. flexneri* 3b infection with a specimen collection date 104 days after the *S. flexneri* 3a infection.

Sixteen DSA isolates were further characterized by NARMS for macrolide resistance genes. All isolates had the *mph*A resistance gene, and 15 isolates had the *erm*B resistance gene.

### Illness Severity and Treatment

We found no major differences in the proportion of patients with fever, bloody diarrhea, or hospitalization among patients with DSA-*Shigella* compared with patients who had azithromycin-susceptible *Shigella*. The median duration of illness was longer for patients with DSA-*Shigella* (11 days) than for patients with azithromycin-susceptible *Shigella* (9 days) (p = 0.004) (Table 3).

**Table 3:** Reported symptoms and illness severity of patients with shigellosis, by azithromycin susceptibility status and CD4 count, Minnesota, USA, 2012–2015*

Characteristic	DSA status, n = 691					CD4 count, cells/mm^3^, n = 878†				
	DSA, n = 46‡	Azithromycin susceptible, n = 64§	OR (95% CI)¶	p value		<200, n = 6	200–500, n = 21	>500, n = 35	Not known to be PLWH, n = 816	p value
Bloody diarrhea	13 (35)	247 (45)	0.7 (0.3–1.3)	0.31		2 (50)	6 (46)	9 (33)	319 (46)	0.65
Fever	23 (66)	406 (74)	0.7 (0.3–1.4)	0.32		3 (75)	9 (69)	18 (69)	525 (75)	0.89
Hospitalized	8 (17)	136 (21)	0.8 (0.4–1.7)	0.71		3 (50)	8 (38)	13 (37)	143 (18)	<0.001#
Death	0	1 (0)	NA	1.00		0	0	0	1 (0)	0.99
Median illness duration, d (range)	11 (3–91)	9 (1–125)	NA	0.004		7 (3–15)	11.5 (4–23)	11 (3–32)	8 (0–125)	0.03**

*Values are no. (%) unless otherwise indicated. DSA, decreased susceptibility to azithromycin; OR, odds ratio; NA, not applicable; PLWH, person living with HIV. †Total number of infections was 882. However, CD4 count was not available for 4 PLWH. ‡Species distribution: *Shigella sonnei*, 52%; *S. flexneri*, 46%; *S. boydii*, 2%. §Species distribution: *S. sonnei*, 83%; *S. flexneri*, 16%; *S. boydii*, 0.6%; *S. dysenteriae*, 0.3%. ¶Reference group is azithromycin-susceptible patients. #Patients not known to be PLWH had lower hospitalization rates. **Patients not known to be PLWH had shorter median duration of illness than PLWH with CD4 counts >200 cells/mm^3^.

Information about antimicrobial drug treatment for shigellosis was available for 752 (85%) infections; 514 (68%) patients were treated with 1 antimicrobial drug and 56 (7%) with multiple antimicrobial drugs. For 182 (24%) infections, the patient either refused treatment (n = 178) or could not recall whether treatment had been given (n = 5). We found similar rates of antimicrobial drug treatment infections with and without DSA (84% vs. 77%; p = 0.54). Patients who received antimicrobial drug treatment reported longer durations of illness (median 9 days vs. 7 days; p = 0.04). Of 103 patients who had antimicrobial susceptibility testing and reported treatment with azithromycin, 4 (4%) had DSA-*Shigella* isolates. Among patients given azithromycin, illness caused by DSA-*Shigella* tended to last longer than that caused by azithromycin-susceptible *Shigella*, but the difference was not significant (median 17 days vs. 7 days; p = 0.06).

### Risk History

Patient interviews were completed for 610 (88%) of the 691 isolates with antimicrobial susceptibility testing results, including 38/46 (82%) with DSA-*Shigella* and 572/645 (88%) that were susceptible to azithromycin. No or few patients with DSA-*Shigella* were exposed to childcare settings (0% vs. 31%; OR undefined; p<0.001) or had children in their home (11% vs. 62%; OR 0.1, 95% CI 0.0–0.2; p<0.001). International travel was reported by the patient or patient’s healthcare provider for 3 (8%) of 39 patients with DSA-*Shigella* and by 66 (12%) of 573 patients with azithromycin-susceptible *Shigella* (OR 0.6, 95% CI 0.2–2.1; p = 0.61).

Travel to Asia was reported by all 3 patients with DSA-*Shigella* infections who reported international travel; 2 were women and 1 was a child. Among those asked, sexual contact with a man in the week before illness onset was reported by 16 (62%) of 26 men with DSA-*Shigella* and 13 (33%) of 40 men with azithromycin-susceptible *Shigella* isolates (OR 3.3, 95% CI 1.2–9.3; p = 0.02). Of the 29 male patients reporting sexual contact with a man, 1 patient with an azithromycin-susceptible infection reported international travel; none reported contact with childcare or children in the household. Among the 66 patients who had sexual contact information on their shigellosis interview and after combining sexual history data from HIV/STD surveillance database (n = 63), 88% of men with DSA-*Shigella* either identified themselves as an MSM during an the HIV/STD interview or reported sexual contact with a man versus 33% of those with azithromycin-susceptible *Shigella*.

No recent STDs were reported among children. Among the 440 isolates collected from adults, 66 (15%) were from PLWH, and 41 (9%) were from patients with a recent STD. Seven (3%) of 223 women were either PLWH (n = 1) or had a recent STD (n = 6); none of these patients had DSA-*Shigella*. Eighty (37%) of 217 isolates collected from men were either from PLWH (n = 65, including 2 patients with 2 *Shigella* infections, and 1 patient with 3 *Shigella* infections) or had a recent STD (n = 36, including 21 patients who were also PLWH). Thirty-two (76%) of 42 men with DSA-*Shigella* were PLWH (n = 25) or had a recent STD (n = 20, including 13 who were also PLWH).

Men with DSA-*Shigella* had greater odds of recent infection with chlamydia (OR 8.3; p<0.001), gonorrhea (OR 5.2; p = 0.001), syphilis (OR 11.7; p = 0.003), any recent STD (OR 9.0; p<0.001), and multiple recent STDs (OR 9.3; p<0.001) compared with men who had susceptible *Shigella* infections (Table 4). In addition, HIV infection was more common among those with DSA-*Shigella* (60% vs. 23%; p<0.001).

**Table 4:** Sexually transmitted diseases reported in the 12 months before *Shigella* specimen collection for 217 men with shigellosis, by azithromycin susceptibility status, Minnesota, USA, 2012–2015*

Disease	DSA, n = 42, no. (%)	Azithromycin susceptible, n = 175, no. (%)	OR (95% CI)†	p value
Chlamydia	13 (31)	9 (5)	8.3 (3.2–21.1)	<0.001
Gonorrhea	10 (24)	10 (6)	5.2 (2.0–13.4)	0.001
Syphilis	5 (12)	2 (1)	11.7 (2.2–62.6)	0.003
HIV‡	25 (60)	40 (23)	5.0 (2.4–10.1)	<0.001
Any bacterial STD§	20 (48)	16 (9)	9.0 (4.1–20.0)	<0.001
Multiple bacterial STDs§	9 (21)	5 (3)	9.3 (2.9–29.4)	<0.001

*All *Shigella* infections reported in men are included. Two men who were PLWH had 2 *Shigella* infections, and 1 man who was PLWH had 3 *Shigella* infections during the study period. DSA, decreased susceptibility to azithromycin; OR, odds ratio; PLWH, persons living with HIV; STD, sexually transmitted disease. †Reference group is men with azithromycin-susceptible *Shigella.* ‡HIV positive at any time before *Shigella* isolation. §Chlamydia, gonorrhea, or syphilis only.

We used a multivariate model that included only men and the variables HIV infection and recent STD. In this model, we found that HIV infection (adjusted OR 3.5, 95% CI 1.6–7.6; p = 0.001) and a recent STD (adjusted OR 6.7, 95% CI 2.8–15.5; p<0.001) were independently associated with DSA-*Shigella*.

Men who were PLWH were not ill longer (median 11 days vs. 9 days; p = 0.10) and not have higher rates of hospitalization (38% vs. 28%; p = 0.14) than other men and did not have higher rates of hospitalization except for PLWH with CD4 counts <200 cells/mm^3^. We found no difference in illness severity among PLWH based on CD4 count. However, patients who were not known to be PLWH had lower rates of hospitalization and shorter median duration of illness than patients who were PLWH with CD4 counts >200 cells/mm^3^ (Table 3). Rates of antimicrobial drug treatment for shigellosis were not higher for PLWH among men (79% vs. 83%; OR 0.8, 95% CI 0.3–1.8; p = 0.66). Isolates from men who were PLWH were more likely than those from other adult males to be MDR (91% vs. 61%; OR 6.4, 95% CI 2.6–15.8; p<0.001), to have clinically relevant resistance (100% vs. 86%; OR undefined; p<0.001), or to be DSA (38% vs. 11%; OR 5.0, 95% CI 2.4–10.1; p<0.001).

## Discussion

We identified DSA among multiple *Shigella* species in Minnesota during 2012–2015. In addition to DSA, we found >1 isolates with resistance to clinically relevant oral antimicrobial drugs and a parenteral agent (ceftriaxone). Increasing resistance to azithromycin has been reported through national antimicrobial drug resistance surveillance every year since 2011 (*26*). However, resistance profiles differed across groups at risk for shigellosis; 42 of the 43 domestically acquired DSA-*Shigella* cases in Minnesota occurred among men, 60% of whom were also infected with HIV. This trend has been observed in other jurisdictions and has implications for clinical testing, public health surveillance, case management, and prevention efforts (*27*–*29*).

DSA-*Shigella* was strongly independently associated with HIV infection and having a recent STD diagnosis. Identifying as an MSM or being a man reporting sexual contact with a man was also associated with DSA-*Shigella* despite the limited data available. Among men with shigellosis, 37% overall and 76% of those with DSA-*Shigella* were either PLWH or had a recent STD. These findings are consistent with other shigellosis outbreak reports among MSM but present more robust estimates of recent STD prevalence among shigellosis patients (*20*,*22*,*27*). Co-infection with STDs is of particular concern because azithromycin is recommended as a treatment for chlamydia, as well as for gonorrhea in conjunction with ceftriaxone (*30*). Nearly half of men with DSA-*Shigella* in this study were given a diagnosis of, and presumably treated for, an STD during the previous year. Treatment of STDs with azithromycin might select for drug-resistant *Shigella* strains circulating in this population. In addition, immunosuppression caused by HIV infection might increase shedding duration and thus the likelihood of *Shigella* transmission (*31*). In our study, severity and duration of illness were not related to CD4 count. However, the sample sizes were small.

Transmission of *Shigella* through sexual contact or activity among MSM was first noted during the 1970s, and outbreaks continue to be documented in the United States and worldwide (*14*,*19*,*32*). Similar to the findings presented in our study, MDR shigellosis has been documented among MSM populations worldwide (*18*,*20*,*33*). Overall, MSM are at greater risk for sexually transmitted *Shigella* infections, and HIV infection can increase the risk for contracting shigellosis (*34*). Efforts to prevent the spread of shigellosis should include outreach to MSM communities to encourage hand and body washing before and after sex, washing sex toys, waiting to have sex until convalescent-phase stool testing confirms that shedding has stopped (or a few weeks if convalescent-phase testing is not performed), asking sexual partners whether they have recently been ill with diarrhea, and use of barriers to prevent fecal–oral contact during sexual activity (*35*).

Additional data about shedding in the context of DSA or HIV infection would help refine this guidance. General shigellosis prevention messages, such as avoiding certain activities such as preparing food for others and swimming while ill, should also be included. In addition, because of the high prevalence of HIV and recent STDs among men with shigellosis, clinicians might use a diagnosis of shigellosis in a man as a reason for HIV and STD screening and use HIV or STD diagnoses to counsel about prevention of shigellosis and other sexually transmitted enteric infections (*29*). Because patients could seek care from clinicians working with MSM in the context of STDs or general practitioners who might not provide treatment for STD patients, cross-training is needed to educate providers for prevention of shigellosis and potential indicators for STD risk.

Shigellosis is typically self-limited and does not require antimicrobial drug treatment, even among PLWH, unless CD4 counts are <500 cells/mm^3^ (*8*). If treatment is deemed necessary because of severity of illness or public health restrictions, antimicrobial susceptibility testing results should be used to guide antimicrobial drug treatment if possible. However, clinical breakpoints for azithromycin resistance in *Shigella* have not been established for clinical laboratories. CLSI recently established epidemiologic cutoff values (ECVs) for azithromycin nonsusceptibility by using MICs for *S. flexneri* and *S. sonnei* and disk diffusion for *S. flexneri* (*25*). In the absence of clinical breakpoints, ECVs are needed for disk diffusion for *S. sonnei.* Although ECVs are useful for epidemiologic purposes, they are not useful for clinical decision making. Azithromycin is a recommended treatment option for shigellosis; therefore, clinical breakpoints are urgently needed. Clinicians should consider antimicrobial drug–resistant shigellosis in MSM patients who have diarrhea, and should specifically request antimicrobial drug susceptibility testing for *Shigella*-positive specimens because such testing is not performed routinely in many clinical laboratories.

In our study, patients with DSA-*Shigella* had a longer duration of illness, but did not experience more severe illness by other measures (i.e., fever, bloody diarrhea, hospitalization). Appropriate antimicrobial drug treatment can shorten the duration of *Shigella* carriage and is often used in an attempt to reduce transmission during outbreaks. However, data about the effectiveness of this strategy are lacking (*1*). Increasing awareness among clinicians is key to increasing stool testing and subsequent antimicrobial susceptibility testing before treatment. Regardless of treatment strategy, adults with shigellosis should be counseled about prevention, including waiting to engage in sexual activity while experiencing and convalescing from diarrhea and using barriers to prevent fecal–oral contact.

In addition to *Shigella* outbreak detection, whole-genome sequencing (WGS) can be used to determine genes that predict resistance to antimicrobial drugs. Most of the DSA isolates tested in our study contained *mph*A and *erm*B genes, both of which are known to confer macrolide resistance (*36*). The increasing use of culture-independent diagnostic testing (CIDT) at clinical laboratories could result in reduced availability of isolates for WGS and susceptibility testing if the capacity to culture CIDT-positive specimens is eliminated from clinical laboratories. To preserve the isolates necessary for determining antimicrobial drug susceptibility and outbreak detection, clinicians are encouraged to order reflex culture for CIDT-positive specimens. Ultimately, isolates should be submitted for antimicrobial susceptibility testing, or WGS if antimicrobial susceptibility testing is not available, to determine antimicrobial drug susceptibility.

Our study findings have limitations. These findings might not be generalizable beyond Minnesota. Case-patients who were given any antimicrobial drugs for shigellosis had longer duration of illness; however, these patients might have received antimicrobial drugs because they had been ill longer, had more severe illness, or had other concurrent conditions. Both data sources are likely incomplete. MSM are probably underreported because both data sources for sexual practices were consistently obtained only during 2015 for shigellosis patients, and sex of sexual partners is not always included on STD or HIV case reports. Also, the period of interest varied between the *Shigella* and HIV/STD case reports, which limited the conclusions that can be drawn from combining those data. Finally, the number of DSA-*Shigella* patients was small, which limited the power of analyses by *Shigella* species.

In conclusion, antimicrobial drug–resistant shigellosis is a growing threat to public health. Treatment recommendations have been modified from ampicillin or trimethoprim/sulfamethoxazole to azithromycin, ciprofloxacin, or ceftriaxone to account for increasing resistance to ampicillin and trimethoprim/sulfamethoxazole (*1*,*8*). However, in Minnesota, prevalence of DSA is increasing among *Shigella* isolates, primarily among adult men, PLWH, and those who have had a recent diagnosis of an STD. Men with *Shigella* infections appear to be at higher risk for MDR; almost 70% of isolates are not susceptible to >3 antimicrobial drug classes. Almost half of men with DSA-*Shigella* infections had a recent STD, indicating that further population-level interventions, such as educational campaigns, are needed to reduce enteric infections spread through sexual activity.

## References

[1] American Academy of Pediatrics. Red book: 2018–2021 report of the committee on infectious diseases. 31st ed. Elk Grove Village (IL): American Academy of Pediatrics; 2018.

[2] Scallan E, Hoekstra RM, Angulo FJ, Tauxe RV, Widdowson M-A, Roy SL, et al. Foodborne illness acquired in the United States—major pathogens. Emerg Infect Dis. 2011;17:7–15. 10.3201/eid1701.P11101 21192848 PMC3375761

[3] Centers for Disease Control and Prevention (CDC). *Shigella flexneri* serotype 3 infections among men who have sex with men—Chicago, Illinois, 2003-2004. MMWR Morb Mortal Wkly Rep. 2005;54:820–2. 16121121

[4] Borg ML, Modi A, Tostmann A, Gobin M, Cartwright J, Quigley C, et al. Ongoing outbreak of *Shigella flexneri* serotype 3a in men who have sex with men in England and Wales, data from 2009-2011. Euro Surveill. 2012;17:20137. 22490381

[5] Drees M, Hathcock AL. Prolonged daycare-associated outbreak caused by *Shigella sonnei*—Delaware, July 2002-April 2003. Del Med J. 2004;76:235–41. 15239384

[6] Morgan O, Crook P, Cheasty T, Jiggle B, Giraudon I, Hughes H, et al. *Shigella sonnei* outbreak among homosexual men, London. Emerg Infect Dis. 2006;12:1458–60. 10.3201/eid1209.060282 17073105 PMC3298285

[7] Shane AL, Tucker NA, Crump JA, Mintz ED, Painter JA. Sharing *Shigella*: risk factors for a multicommunity outbreak of shigellosis. Arch Pediatr Adolesc Med. 2003;157:601–3. 10.1001/archpedi.157.6.601-a 12796243

[8] US Department of Health and Human Services. Guidelines for the prevention and treatment of opportunistic infections in HIV-infected adults and adolescents, August 10, 2017 [cited 2018 Oct 4]. https://aidsinfo.nih.gov/guidelines/html/4/adult-and-adolescent-opportunistic-infection/328/bacterial-enteric

[9] Choi S-H, Kim EY, Kim Y-J. Systemic use of fluoroquinolone in children. Korean J Pediatr. 2013;56:196–201. 10.3345/kjp.2013.56.5.196 23741232 PMC3668199

[10] Valcanis M, Brown JD, Hazelton B, OʼSullivan MV, Kuzevski A, Lane CR, et al. Outbreak of locally acquired azithromycin-resistant *Shigella flexneri* infection in men who have sex with men. Pathology. 2015;47:87–8. 10.1097/PAT.0000000000000207 25474524

[11] Bowen A, Grass J, Bicknese A, Campbell D, Hurd J, Kirkcaldy RD. Elevated risk for antimicrobial drug–resistant *Shigella* infection among men who have sex with men, United States, 2011–2015. Emerg Infect Dis. 2016;22:1613–6. 10.3201/eid2209.160624 27533624 PMC4994375

[12] Rahman M, Shoma S, Rashid H, El Arifeen S, Baqui AH, Siddique AK, et al. Increasing spectrum in antimicrobial resistance of *Shigella* isolates in Bangladesh: resistance to azithromycin and ceftriaxone and decreased susceptibility to ciprofloxacin. J Health Popul Nutr. 2007;25:158–67. 17985817 PMC2753991

[13] Howie RL, Folster JP, Bowen A, Barzilay EJ, Whichard JM. Reduced azithromycin susceptibility in *Shigella sonnei*, United States. Microb Drug Resist. 2010;16:245–8. 10.1089/mdr.2010.0028 20624094

[14] Sjölund Karlsson M, Bowen A, Reporter R, Folster JP, Grass JE, Howie RL, et al. Outbreak of infections caused by *Shigella sonnei* with reduced susceptibility to azithromycin in the United States. Antimicrob Agents Chemother. 2013;57:1559–60. 10.1128/AAC.02360-12 23274665 PMC3591876

[15] Gaudreau C, Barkati S, Leduc JM, Pilon PA, Favreau J, Bekal S. *Shigella* spp. with reduced azithromycin susceptibility, Quebec, Canada, 2012-2013. Emerg Infect Dis. 2014;20:854–6. 10.3201/eid2005.130966 24750584 PMC4012797

[16] Heiman KE, Grass JE, Sjölund-Karlsson M, Bowen A. Shigellosis with decreased susceptibility to azithromycin. Pediatr Infect Dis J. 2014;33:1204–5. 10.1097/INF.0000000000000397 25361413 PMC4700834

[17] Boumghar-Bourtchai L, Mariani-Kurkdjian P, Bingen E, Filliol I, Dhalluin A, Ifrane SA, et al. Macrolide-resistant *Shigella sonnei.* Emerg Infect Dis. 2008;14:1297–9. 10.3201/eid1408.080147 18680661 PMC2600399

[18] Baker KS, Dallman TJ, Ashton PM, Day M, Hughes G, Crook PD, et al. Intercontinental dissemination of azithromycin-resistant shigellosis through sexual transmission: a cross-sectional study. Lancet Infect Dis. 2015;15:913–21. 10.1016/S1473-3099(15)00002-X 25936611

[19] Bowen A, Eikmeier D, Talley P, Siston A, Smith S, Hurd J, et al.; Centers for Disease Control and Prevention (CDC). Notes from the field: outbreaks of *Shigella sonnei* infection with decreased susceptibility to azithromycin among men who have sex with men—Chicago and metropolitan Minneapolis-St. Paul, 2014. MMWR Morb Mortal Wkly Rep. 2015;64:597–8. 26042652 PMC4584772

[20] Heiman KE, Karlsson M, Grass J, Howie B, Kirkcaldy RD, Mahon B, et al.; Centers for Disease Control and Prevention (CDC). Notes from the field: Shigella with decreased susceptibility to azithromycin among men who have sex with men - United States, 2002-2013. MMWR Morb Mortal Wkly Rep. 2014;63:132–3. 24522098 PMC4584870

[21] Danila RN, Eikmeier DL, Robinson TJ, La Pointe A, DeVries AS. Two concurrent enteric disease outbreaks among men who have sex with men, minneapolis-st paul area. Clin Infect Dis. 2014;59:987–9. 10.1093/cid/ciu478 24944234

[22] Gilbart VL, Simms I, Jenkins C, Furegato M, Gobin M, Oliver I, et al. Sex, drugs and smart phone applications: findings from semistructured interviews with men who have sex with men diagnosed with *Shigella flexneri* 3a in England and Wales. Sex Transm Infect. 2015;91:598–602. 10.1136/sextrans-2015-052014 25921020

[23] Gaudreau C, Helferty M, Sylvestre JL, Allard R, Pilon PA, Poisson M, et al. *Campylobacter coli* outbreak in men who have sex with men, Quebec, Canada, 2010-2011. Emerg Infect Dis. 2013;19:764–7. 10.3201/eid1905.121344 23647786 PMC3647503

[24] Simms I, Gilbart VL, Byrne L, Jenkins C, Adak GK, Hughes G, et al. Identification of verocytotoxin-producing *Escherichia coli* O117:H7 in men who have sex with men, England, November 2013 to August 2014. Euro Surveill. 2014;19:20946. 10.2807/1560-7917.ES2014.19.43.20946 25375900

[25] Clinical and Laboratory Standards Institute. Performance standards for antimicrobial susceptibility testing. 29th ed CLSI Supplement M100S. Wayne (PA): The Institute; 2019. p. 249.

[26] Centers for Disease Control and Prevention. National antimicrobial resistance monitoring system for enteric bacteria (NARMS): human isolates surveillance report for 2015 (final report) [cited 2020 Jan 9]. https://www.cdc.gov/narms/reports/annual-human-isolates-report-2015.html

[27] Murray K, Reddy V, Kornblum JS, Waechter H, Chicaiza LF, Rubinstein I, et al. Increasing antibiotic resistance in *Shigella* spp. from infected New York City residents, New York, USA. Emerg Infect Dis. 2017;23:332–5. 10.3201/eid2302.161203 28098543 PMC5324786

[28] Mohan K, Hibbert M, Rooney G, Canvin M, Childs T, Jenkins C, et al. What is the overlap between HIV and shigellosis epidemics in England: further evidence of MSM transmission? Sex Transm Infect. 2018;94:67–71. 10.1136/sextrans-2016-052962 28490580

[29] Goulart MA, Wurcel AG. Shigellosis in men who have sex with men: an overlooked opportunity to counsel with pre-exposure prophylaxis for HIV. Int J STD AIDS. 2016;27:1236–8. 10.1177/0956462416638609 26945593

[30] Workowski KA, Bolan GA; Centers for Disease Control and Prevention. Sexually transmitted diseases treatment guidelines, 2015. MMWR Recomm Rep. 2015;64(RR-03):1–137. 26042815 PMC5885289

[31] Daskalakis DC, Blaser MJ. Another perfect storm: *Shigella*, men who have sex with men, and HIV. Clin Infect Dis. 2007;44:335–7. 10.1086/510591 17205437

[32] Dritz SK, Back AF. Letter: *Shigella* enteritis venereally transmitted. N Engl J Med. 1974;291:1194. 10.1056/NEJM197411282912223 4608062

[33] Gaudreau C, Ratnayake R, Pilon PA, Gagnon S, Roger M, Lévesque S. Ciprofloxacin-resistant *Shigella sonnei* among men who have sex with men, Canada, 2010. Emerg Infect Dis. 2011;17:1747–50. 10.3201/eid1709.102034 21888811 PMC3322076

[34] Aragón TJ, Vugia DJ, Shallow S, Samuel MC, Reingold A, Angulo FJ, et al. Case-control study of shigellosis in San Francisco: the role of sexual transmission and HIV infection. Clin Infect Dis. 2007;44:327–34. 10.1086/510593 17205436

[35] Centers for Disease Control and Prevention. *Shigella* infections among gay and bisexual men, 2016 [cited 2019 Oct 21]. https://www.cdc.gov/shigella/msm.html

[36] Yousfi K, Gaudreau C, Pilon PA, Lefebvre B, Walker M, Fournier É, et al. Genetic mechanisms behind the spread of reduced susceptibility to azithromycin in *Shigella* strains isolated from men who have sex with men in Québec, Canada. Antimicrob Agents Chemother. 2019;63:e01679–18. 30455248 10.1128/AAC.01679-18 PMC6355565

